# Aging and Neurodegenerative Disease: Is the Adaptive Immune System a Friend or Foe?

**DOI:** 10.3389/fnagi.2020.572090

**Published:** 2020-09-23

**Authors:** Katie Mayne, Jessica A. White, Christopher E. McMurran, Francisco J. Rivera, Alerie G. de la Fuente

**Affiliations:** ^1^Wellcome-Wolfson Institute for Experimental Medicine, School of Medicine, Dentistry and Biomedical Science, Queen’s University Belfast, Belfast, United Kingdom; ^2^Department of Medicine, Addenbrooke’s Hospital, Cambridge, United Kingdom; ^3^Laboratory of Stem Cells and Neuroregeneration, Institute of Anatomy, Histology and Pathology, Faculty of Medicine, Universidad Austral de Chile, Valdivia, Chile; ^4^Center for Interdisciplinary Studies on the Nervous System (CISNe), Universidad Austral de Chile, Valdivia, Chile; ^5^Institute of Molecular Regenerative Medicine, Paracelsus Medical University, Salzburg, Austria; ^6^Spinal Cord Injury and Tissue Regeneration Center Salzburg (SCI-TReCS), Paracelsus Medical University, Salzburg, Austria

**Keywords:** aging, adaptive immune system, neurodegenerative diseases, degeneration, regeneration

## Abstract

Neurodegenerative diseases of the central nervous system (CNS) are characterized by progressive neuronal death and neurological dysfunction, leading to increased disability and a loss of cognitive or motor functions. Alzheimer’s disease, Parkinson’s disease and amyotrophic lateral sclerosis have neurodegeneration as a primary feature. However, in other CNS diseases such as multiple sclerosis, stroke, traumatic brain injury, and spinal cord injury, neurodegeneration follows another insult, such as demyelination or ischaemia. Although there are different primary causes to these diseases, they all share a hallmark of neuroinflammation. Neuroinflammation can occur through the activation of resident immune cells such as microglia, cells of the innate and adaptive peripheral immune system, meningeal inflammation and autoantibodies directed toward components of the CNS. Despite chronic inflammation being pathogenic in these diseases, local inflammation after insult can also promote endogenous regenerative processes in the CNS, which are key to slowing disease progression. The normal aging process in the healthy brain is associated with a decline in physiological function, a steady increase in levels of neuroinflammation, brain shrinkage, and memory deficits. Likewise, aging is also a key contributor to the progression and exacerbation of neurodegenerative diseases. As there are associated co-morbidities within an aging population, pinpointing the precise relationship between aging and neurodegenerative disease progression can be a challenge. The CNS has historically been considered an isolated, “immune privileged” site, however, there is mounting evidence that adaptive immune cells are present in the CNS of both healthy individuals and diseased patients. Adaptive immune cells have also been implicated in both the degeneration and regeneration of the CNS. In this review, we will discuss the key role of the adaptive immune system in CNS degeneration and regeneration, with a focus on how aging influences this crosstalk.

## Introduction

Neurodegenerative disease defines conditions in which there is progressive neuronal loss in the central nervous system (CNS), leading to either physical disability, cognitive deficits or both. Classical neurodegenerative diseases in which neurodegeneration is the key hallmark includes Alzheimer’s disease (AD), Parkinson’s disease (PD), and amyotrophic lateral sclerosis (ALS) (Dugger and Dickson, 2017). However, other diseases can be defined as neurodegenerative when a primary insult such as demyelination, ischaemia or trauma leads to neuronal loss. Multiple sclerosis (MS), stroke and traumatic injury to the CNS are all examples of secondary neurodegenerative diseases (Amor et al., 2010). Aging is a major risk factor for neurodegenerative disease, and with a growing elderly population, its prevalence is continuously increasing (Wyss-Coray, 2016). Beyond being a risk factor, aging also increases the severity of disease and results in an impaired recovery following insult. Although these diseases have different pathogenetic mechanisms such as protein aggregation, demyelination, ischaemia, or direct trauma, they all share a hallmark of neuroinflammation (Stephenson et al., 2018).

The immune system plays a key role in CNS homeostasis and disease. The innate immune system is the first line of defense against pathogens (Chaplin, 2010) and CNS-resident macrophages, microglia, are of vital importance as early respondents to CNS alterations such as damage or infection but also in development and homeostasis (Bachiller et al., 2018). Microglia activation is also an important component of neuroinflammation, aging, and different neurodegenerative diseases either directly via phagocytosis and cytokine production, as shown by the identification of disease-specific microglia, or indirectly in response to cues from the adaptive immune system (Keren-Shaul et al., 2017; Deczkowska et al., 2018). The adaptive immune system is an important component of the host defense against pathogens, through the recognition of non-self antigens (Chaplin, 2010). This defensive mechanism is mediated by B and T lymphocytes which display a diverse range of specific antigen receptors during humoral and cellular-mediated immunity (Chaplin, 2010). Although the CNS was once considered an ‘immune-privileged’ site, recent studies have indicated the presence and importance of the adaptive immune system in the CNS for immune-surveillance and defense against neurotropic viruses (Ellwardt et al., 2016). Studies have also highlighted the role of adaptive immunity in maintaining CNS homeostasis and integrity, promoting neurogenesis and improving cognitive function (Ziv et al., 2006; Brynskikh et al., 2008; Radjavi et al., 2014). In healthy individuals, this immune-CNS interaction is highly regulated to maintain the beneficial relationship. However, during both aging and neurodegenerative disease, the blood-brain barrier (BBB) is disrupted, leading to an increased infiltration of peripheral immune cells into the CNS, where they can potentiate further neurodegeneration or facilitate tissue regeneration. In both neurodegenerative disease and the normal aging process, there is a common theme of immune dysregulation and abnormal immune responses. This review will discuss the involvement of the adaptive immune system in neurodegenerative disease, highlighting its role in degeneration and regeneration, and the impact of aging in disease pathogenesis.

## Healthy CNS

In addition to the key role of the adaptive immune system in CNS homeostasis and immunosurveillance, it also influences brain development and behavior, particularly affecting hippocampal neurogenesis. Severe combined immunodeficient (SCID) mice, lacking mature lymphocytes, showed impaired neurogenesis compared to wild-type (WT) mice, in which T cells infiltrate the CNS (Ziv et al., 2006). SCID mice also show a reduced learning capacity and impaired memory (Brynskikh et al., 2008; Luo et al., 2019), possibly associated with a higher expression of neurotransmission-related genes, indicating dysfunctional synaptic connectivity or an imbalance of neurotransmitters as a result of lymphocyte deficiency (Luo et al., 2019). Adaptive immune cells in particular are important for normal learning and memory function, as Rag2^–/–^ mice, which lack mature lymphocytes, show impaired cognitive function (Radjavi et al., 2014). This effect is T cell-mediated as function was restored upon adoptive transfer of splenocytes but not T cell-depleted splenocytes (Kipnis et al., 2004; Ziv et al., 2006; Brynskikh et al., 2008). Similarly, nude mice lacking mature lymphocytes show diminished neurogenesis and impaired cognitive function (Kipnis et al., 2004; Ziv et al., 2006). T cell activation and a simultaneous increase in corticosterone levels also leads to a transient increase in proliferation of hippocampal progenitor cells and neurogenesis (Wolf et al., 2009b).

Adoptive transfer of CD3^+^, CD4^+^, and CD8^+^ T cells derived from human umbilical cord blood mononuclear cells into rats was shown to enhance the proliferation and survival of neural stem cells (Shahaduzzaman et al., 2013). However, other studies suggest that CD4^+^ T cells, but not CD8^+^ T cells or B cells, are involved in promoting hippocampal neurogenesis and maintaining cognitive function, as only CD4^+^ T cell depletion impaired these processes (Wolf et al., 2009a). Even if the role for CD4^+^ T cells in neurogenesis is widely accepted, the involvement of antigen-specificity has been debated. Wolf et al. suggest a systemic mechanism of CD4^+^ T cell-mediated neurogenesis, showing that repopulation of Rag2^–/–^ mice with non-specific CD4^+^ T cells increases neural stem cell proliferation (Wolf et al., 2009a). Others, however, have shown the requirement for CNS antigen specificity, as increased neurogenesis and improved cognitive function was only observed with the adoptive transfer of MBP-specific T cells and not ovalbumin (OVA)-specific T cells, reactive toward a foreign protein (Ziv et al., 2006). Mice which only have OVA-specific T cells present learning and memory deficits, which were restored upon transfer of MOG-specific CD4^+^ T cells (Radjavi et al., 2014). Nonetheless, both studies agreed on the CD4^+^ T cell-mediated promotion of brain-derived neurotrophic factor (BDNF), which is required for neuronal survival and differentiation (Ziv et al., 2006; Wolf et al., 2009a).

**FIGURE 1:**
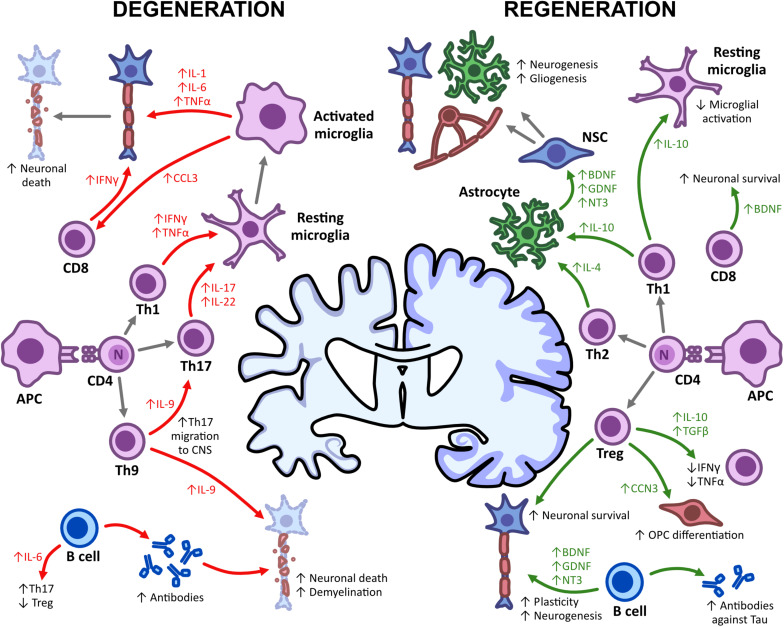
Adaptive immunity in CNS degeneration and regeneration. The damage and repair of the CNS is mediated by immune mechanisms, with both T and B cells having detrimental and regenerative effects. Upon APC-T lymphocyte interactions, T effector cells such as Th1, Th17, and Th9 promote microglial activation through the expression of pro-inflammatory cytokines (IFN-γ, TNFα, IL-9, and IL-17). This subsequently enhances neuronal death and demyelination and leads to a decline in cognitive functioning. Th1, Th2, and Treg cells, however, can also promote regeneration, enhancing neurogenesis, gliogenesis and remyelination upon the secretion of IL-4, IL-10, and TGFβ. Somewhat similar to Th1, CD8^+^ T cells, and B cells also increase neuronal death but can promote survival through neurotrophic production (BDNF, GDNF, and NT3) and an increased antibody repertoire. This shows the impact of immune-mediated mechanisms on degeneration/regeneration and some possible targets for immunotherapies.

In contrast, neurogenesis is impacted by aging, where neurogenic niches become dysfunctional, in part due to an increased infiltration of CD8^+^ T cells (Dulken et al., 2019). Gene expression changes in CD8^+^ T cells and neuronal stem cells in aged neurogenic niches show increased genes related to the IFN-γ pathway and IFN-γ signaling, respectively (Dulken et al., 2019). This highlights the dynamic balance between the beneficial and detrimental impacts of the adaptive immune system in the CNS, depending on the environment (summarized in Figure 1). The interaction between the CNS, adaptive immune system and aging is further modified by disease states. We next address these relationships in the context of several common neurodegenerative diseases (summarized in Tables 1, 2).

**TABLE 1:** Contribution of the adaptive immune system in primary neurodegenerative diseases.

**Primary neurodegenerative diseases**	**Neurodegeneration**	**Regeneration**
Alzheimer’s disease	Rag-5xfAD mice show enhanced Aβ pathology and neuroinflammation (Marsh et al., 2016).	AB-specific Th2 cells promote neurological recovery (Cao et al., 2009).
	IFN-y producing Th1 cells enhance microglial activation and Aβ deposition (McQuillan et al., 2010; Browne et al., 2013).	IL-17 depletion enhances neural precursor cell expression and synaptic transmission (Liu et al., 2014).
	Depletion of Tregs in mice accelerates AD-related cognitive dysfunction (Baek et al., 2016; Dansokho et al., 2016).	Adoptive transfer of Tregs reduces Aβ deposition and reverses cognitive deficits (Baek et al., 2016).
Parkinson’s disease	SCID, Rag1 KO, TCR B KO, CD4 KO but not CD8 KO mice show attenuated dopaminergic cell death (Benner et al., 2008; Brochard et al., 2009; Gonzalez et al., 2013).	Copolymer 1 immunized T cells administered to MPTP mice limited neuronal loss (Schori et al., 2001; Benner et al., 2004).
	Adoptive transfer of T cells from mice immunized with α-synuclein exacerbate MTPT (Reynolds et al., 2010).	Adoptive transfer of activated Tregs to MPTP provides 90% neuronal protection (Reynolds et al., 2007).
	IL-17 increases cell death in iPSC-derived neurons from PD patients (Sommer et al., 2019).	GM-CSF administration increases Tregs, limits inflammation and increases neuroprotection (Kosloski et al., 2013; Gendelman et al., 2017; Schutt et al., 2018).
	Suppression of CD4 T cell infiltration ameliorates PD symptoms (Qin et al., 2016).	
Amyotrophic lateral sclerosis	A Th1/Th17 immune response correlates with disease progression and severity (Saresella et al., 2013; Jin et al., 2020).	Reconstitution with CD4^+^ T cells in SOD1G93A mice increases neuroprotection (Beers et al., 2008).
	In the SOD1^*G93A*^ model, CD8^+^ T cell ablation leads to a reduction in motor neuron loss (Coque et al., 2019).	Disease progression reduces Tregs (Beers et al., 2011).
	A2BG2 glycan is increased in IgG antibodies for SOD1G93A mice, increasing neuronal cytotoxicity and death (Edri-Brami et al., 2015).	Adoptive transfer of activated Tregs to SOD1G93A mice delays motor function loss and enhances survival (Banerjee et al., 2008).

**TABLE 2:** Contribution of the adaptive immune system in neurodegeneration and regeneration secondary to other pathology.

**Secondary neurodegenerative diseases**	**Neurodegeneration**	**Regeneration**
Multiple sclerosis	T cell depleted mice do not develop EAE (Ortiz-ortiz and Weigle, 1976). Adoptive transfer of MBP-specific CD4^+^T cells induce EAE (Pettinelli and McFarlin, 1981; Zamvil et al., 1985). Adoptive transfer of Th1 and Th17 cells induce classical and atypical EAE (Jager et al., 2009; Domingues et al., 2010).	Mice deficient in CD4^+^ or CD8^+^ T cells show impaired remyelination following lysolecithin-induced demyelination (Bieber et al., 2003). Tregs promote OPC differentiation and efficient remyelination (Dombrowski et al., 2017).
Stroke	Rag1 KO and SCID mice show a reduced infarct size after MCAO (Hurn et al., 2007; Kleinschnitz et al., 2010). Adoptive transfer of CD8 T cells into Rag1 KO mice increases infarct size (Mracsko et al., 2014). Specific antibody-mediated depletion of either CD4^+^ or CD8^+^ T cells decreased infarct size (Liesz et al., 2011). B lymphocytes mediate a delayed cognitive impairment following stroke in mice (Doyle et al., 2015).	Tregs accumulate following ischaemia and have a role in suppressing astrogliosis and promoting neurological recovery (Ito et al., 2019). Administration of CD34^+^ immune progenitor cells promoted revascularisation and neurogenesis (Taguchi et al., 2004).
Traumatic CNS injury	**In SCI:** SCID mice show better functional recovery after SCI (Luo et al., 2019). Rag2 KO mice, BCKO mice, Athymic nude rats and B cell depletion show less degeneration and improved recover (Potas et al., 2006; Ankeny et al., 2009; Wu et al., 2012; Casili et al., 2016). MBP-reactive lymphocytes contribute to SCI neurodegeneration (Jones et al., 2002). Antibodies against CXCL10 ameliorate SCI (Gonzalez et al., 2003). Rag1 KO mice administered with IgM antibodies show exacerbated pathology (Narang et al., 2017). CD8^+^ T cells inhibit neurite outgrowth *in vitro* (Pool et al., 2012). **In TBI:** Rag1^–/–^ mice have a similar injury extent to controls in the closed head injury model (Weckbach et al., 2012). Inhibition of antigen processing/presentation reduces lesion size in a fluid percussion trauma model (Tobin et al., 2014).	**In SCI:** CD4 T cells promote neurite outgrowth *in vitro* (Pool et al., 2012). Adoptive transfer of Th1 cells promotes locomotor recovery in SCI (Ishii et al., 2012). Adoptive transfer of CD4^+^ T cells into IL-4 deficient mice promotes neuronal survival and regeneration (Walsh et al., 2015). Active immunization with MBP or transfer of MBP-T cells enhance SCI locomotor recovery and regeneration (Hauben et al., 2000). Myelin and spinal cord homogenate immunization improves regeneration and recovery (Huang et al., 1999; Sicotte et al., 2003). **In TBI:** Vaccination with Cop-1 (a synthetic mimic of MBP epitopes) reduced neuronal loss and promoted recovery (Kipnis et al., 2003).

## Alzheimer’s Disease

Alzheimer’s disease is a progressive neurodegenerative disease with key hallmarks of cognitive dysfunction, memory loss and behavioral disturbances in the elderly population (Chen and Mobley, 2019). AD is primarily characterized by the presence of amyloid beta (Aβ) plaques and neurofibrillary tangles (NFT) of hyperphosphorylated tau in the brain (Chen and Mobley, 2019), leading to synaptic loss, reduced dendritic spines and neuronal death (Paulson et al., 2008). These protein aggregates not only cause neurodegeneration but also lead to the dysfunction of other glial cells such as oligodendrocytes, astrocytes, and microglia (Jantaratnotai et al., 2003; Desai et al., 2011). Accumulation is also associated with microglial and astrocyte activation, which induces inflammation and oxidation, promoting further neuronal dysfunction and apoptosis (Hardy and Allsop, 1991; Kametani and Hasegawa, 2018). Age is the most important risk factor for AD, with ∼90% of cases being late-onset and ∼10% early-onset (Tanzi, 2012; Bature et al., 2017). However, genetics is also a risk factor, particularly in early-onset patients who are more likely to have familial AD with mutations in the Aβ precursor protein (APP) and the presenilin genes (PSEN1 and PSEN2) (Bature et al., 2017; Chen and Mobley, 2019).

Neuroinflammation has been implicated in the pathogenesis of AD, with the innate immune system thought to play a dominating role in the recruitment of microglia to the site of damage (Nordengen et al., 2019). However, the role of the adaptive immune system in AD remains poorly understood. Evidence has shown increased numbers of T lymphocytes in the post-mortem brain tissue of AD patients compared to healthy controls (Rogers et al., 1988; Togo et al., 2002). The increased number of CD3^+^ T cells in AD patients brains were mostly CD8^+^ T cells, which significantly correlated with tau but not Aβ burden, suggesting a role for T cells in NFT development (Merlini et al., 2018). In an animal model of AD, in which mutations in APP and PS1 cause elevated levels of Aβ, there was similarly an increase of T cells in the brain parenchyma (Browne et al., 2013). Evaluation of T cell subsets in the peripheral blood of AD patients showed decreased regulatory T cells (Tregs) (Ciccocioppo et al., 2019), increased Th17 cells (Oberstein et al., 2018) and increased CD8^+^ T cells (Gate et al., 2020). Another study showed similar proportions of Tregs in the peripheral blood of patients and controls, and established a correlation between circulating Tregs and tau in the CSF as an indication of less severe disease (Oberstein et al., 2018).

Aβ-specific Th1 cells adoptively transferred into APP/PS1 mice leads to microglial activation, production of pro-inflammatory cytokines, Aβ deposition and impaired cognitive function (Browne et al., 2013). This pathogenic effect is thought to be mediated by IFN-γ, with IFN-γ neutralization attenuating the Th1-associated detrimental effect (Browne et al., 2013). However, a contradictory study shows an IFN-γ-producing CD4^+^ T cell-dependent mechanism of macrophage recruitment and Aβ clearance, leading to improved cognitive function in an AD mouse model (5xFAD) (Baruch et al., 2016). These contradictions may be due to the different methods used, with one transferring Aβ-specific Th1 cells and the other inducing a natural increase in Th1 cells due to treatment with a PD-1 immune checkpoint inhibitor. Induction of AD in rats through the injection of Aβ into the hippocampus also causes Th17 cell infiltration and the upregulation of IL-17 and IL-22 in the hippocampus, blood and CSF (Zhang J. et al., 2013). Th17 cells are similarly thought to promote neurodegeneration by acting directly on neurons via the Fas/FasL apoptotic pathway (Zhang J. et al., 2013) and are also implicated in the dysfunctional neurogenesis seen in AD; which can be rescued by the genetic deletion of IL-17 (Liu et al., 2014).

There is further evidence of CD8^+^ T cell trafficking in AD, with clonally expanded antigen-specific CD8^+^ T cells in the CSF of AD patients (Gate et al., 2020). In THY-Tau22 mice, which develop tau pathology and cognitive dysfunction, activation of microglia and astrocytes was accompanied by an infiltration of CD8^+^ T cells linked to an early CCL3 chemokine response (Laurent et al., 2017). Similarly, CD8^+^ T cell infiltration was also observed in AD patients with a P301L tau mutation (Laurent et al., 2017). T cell depletion reversed cognitive deficits without affecting tau burden, indicating a detrimental T cell response to tau burden (Laurent et al., 2017). These studies therefore support a role of CD8^+^ T cells in neuroinflammation and neurodegeneration in a model of tauopathy.

Although the adaptive immune system, specifically T lymphocytes, have been implicated in AD pathogenesis, the presence of these cells can also be beneficial. Adaptive immune cells were genetically deleted in the 5xFAD animal model, enhancing AD pathology (Rag-5xFAD) (Marsh et al., 2016). Rag-5xFAD mice showed increased Aβ pathology and neuroinflammation, following the upregulation of pro-inflammatory cytokines implicated in neurodegeneration, plaque formation and cognitive impairments (IL-1β, IL-6, and TNFα) (Cacabelos et al., 1994; Huell et al., 1995; Tweedie et al., 2012; Marsh et al., 2016). Bone marrow transplantation to replace the adaptive immune cells negated the AD pathology, mainly through microglia modulation (Marsh et al., 2016).

Research into T cell subsets has highlighted a role for Th2 cells and Tregs in the reduction of AD pathology and regeneration. Aβ-specific polarized Th2 cells adoptively transferred into APP/PS1 mice showed reduced plaque-associated microglia, reduced Aβ deposits, and a reversal of cognitive dysfunction, with a memory and identification ability recovery similar to WT animals (Cao et al., 2009). Th2-mediated reversal of AD pathology may be IL-4-dependent, as IL-4^–/–^ mice show cognitive impairments which are reversed upon the adoptive transfer of WT T cells (Derecki et al., 2010). This is further supported by studies showing that T cell-derived IL-4 can modulate meningeal myeloid cells, increasing astrocyte expression of BDNF, associated with reduced cognitive deficits and neurodegeneration in a mouse model of tauopathy (Derecki et al., 2010; Jiao et al., 2016).

Tregs delay the progression of AD pathology, with Treg depletion accelerating cognitive decline (Baek et al., 2016; Dansokho et al., 2016). Increasing Treg numbers reversed the cognitive deficits observed in APP/PS1 mice and increased microglial numbers in plaques (Dansokho et al., 2016). Adoptive transfer of Tregs into 3xTg-AD mice similarly improved cognitive function and reduced Aβ deposition and plaque formation (Baek et al., 2016). Although total depletion is detrimental, the transient genetic ablation of Tregs resulted in improved cognitive functioning and a reduction in Aβ plaque area, through an increased infiltration of macrophages and CD4^+^ T cells (Baruch et al., 2015). This suggests that controlled Treg depletion may be neuroprotective and instead contribute to AD mitigation by minimizing the suppression of other T cell subsets.

## Parkinson’s Disease

Parkinson’s disease is another example of a classical neurodegenerative disease of the CNS, characterized by motor dysfunction and neuropsychiatric symptoms. The hallmarks of PD pathogenesis are Lewy body/neurite formation and death of dopamine-secreting neurons in the substantia nigra, a region which modulates motor movement (Eriksen et al., 2005). Lewy bodies and neurites are formed by the aggregation of α-synuclein within neuronal cell bodies and neuronal processes, respectively (Eriksen et al., 2005). Although the cause of PD is unknown, there are hypotheses of disease pathogenesis including neuroinflammation, mitochondrial defects and dysfunctional protein clearance (DeMaagd and Philip, 2015). Neuroinflammation in PD is a result of activated microglia and monocytes in response to misfolded protein α-synuclein (Mosley et al., 2012). These innate immune cells secrete pro-inflammatory and neurotoxic cytokines and chemokines, leading to BBB disruption and the infiltration of lymphocytes to the site of damage (Mosley et al., 2012). Although evidence for the role of the immune system can be distinguished through blood and post-mortem brain samples, and through the efficacy of treatment trials, much of the research refers to the 1-methyl-4-phenyl-1,2,3,6-tetrahydropyridine (MPTP) PD mouse model. This model employs a toxin-induced death of dopaminergic neurons to mimic the damage seen in PD patient brains (Meredith and Rademacher, 2011).

Systemic inflammation increases the risk of PD (Chen et al., 2003), however, the numbers of lymphocytes within the periphery and the CNS remains controversial. Studies have shown a decrease in T and B lymphocytes in the peripheral blood of PD patients (Bas et al., 2001; Baba et al., 2005; Stevens et al., 2012). While there are fewer B lymphocytes, the levels of α-synuclein-specific autoantibodies are increased in the blood and CSF of PD patients (Horvath et al., 2017; Shalash et al., 2017; Akhtar et al., 2018). The decreased number of B lymphocytes is also associated with alterations in the expression of B cell-related genes in peripheral blood leukocytes in PD patients (Kobo et al., 2016). However, it is unclear whether the change in number or functionality of B lymphocytes are causal or secondary to CNS injury in PD (Sabatino et al., 2019). Evaluation of T lymphocyte subsets showed a decrease in CD4^+^ T cells with an increase in CD8^+^ T cells (Baba et al., 2005; Saunders et al., 2012; Stevens et al., 2012). Despite this decrease in CD4^+^ T cells, there is an evident shift in cell phenotype proportions, with decreased naïve and increased effector and memory cells (Fiszer et al., 1994; Bas et al., 2001; Saunders et al., 2012). There is further controversy on the proportions of CD4^+^ T cell subsets in the circulation of PD patients, as one study highlights an increase in Tregs (Bas et al., 2001) and others show a decrease and loss in functionality (Baba et al., 2005; Saunders et al., 2012; Chen et al., 2015; Sommer et al., 2019). Levels of Th1 cells are also debated, some studies show a shift toward a Th1 immune response with IFN-γ production, correlating with disease rating scores (Baba et al., 2005; Chen et al., 2015). Others, however, show decreased Th1 cells and no evidence of subset dominance in PD patients (Niwa et al., 2012; Sommer et al., 2019). An increase in Th17 cells has also been shown in the peripheral blood of PD patients (Chen et al., 2015; Sommer et al., 2019). While these studies do not always agree on lymphocyte levels, there is consistent evidence for immune cell dysregulation in the circulation of PD patients. CD3^+^ T cells infiltrate the brains of PD patients, with post-mortem brain tissue showing cells localized around damaged neurons (Sommer et al., 2019). The immune infiltrate consists of both CD4^+^ and CD8^+^ T cells, but not B cells, suggesting a role for T lymphocytes in the pathogenesis or repair of PD (Brochard et al., 2009). Although B cells were not identified in PD post-mortem tissue, another study showed IgG deposits on Lewy bodies and dopaminergic neurons, highlighting a role for B cell antibody production in PD (Orr et al., 2005).

Neuroinflammation has proven to be pathogenic in PD through inhibition of the JAK/STAT pathway, which is critical in the modulation of the immune system (Qin et al., 2016). Inhibiting the JAK/STAT pathway in rats overexpressing α-synuclein prevented the loss of dopaminergic neurons and neuroinflammation, following the suppression of both microglial activation and CD4^+^ T cell infiltration (Qin et al., 2016). Lymphocytes are also implicated in the loss of dopaminergic neurons, with recent investigations suggesting a potential autoimmune role for T lymphocytes in PD, as patient-derived T cells recognize α-synuclein in pre-clinical and early PD cases (Sulzer et al., 2017; Lindestam Arlehamn et al., 2020).

The role of the adaptive immune system in the pathogenesis of PD was shown using different MPTP models. In an MPTP monkey model of PD, treatment with the antiviral oral drug Maraviroc led to a reduced infiltration of T lymphocytes into the CNS, protecting from nigrostriatum neuronal cell death and improving locomotor activities (Mondal et al., 2019). Similarly, SCID, Rag1^–/–^ and Tcrb^–/–^ mice, all lacking mature lymphocytes, show attenuated dopaminergic cell death following MPTP treatment, an effect abolished upon reconstitution with WT splenocytes (Benner et al., 2008; Brochard et al., 2009). This detrimental effect is mediated by CD4^+^ T cells, as CD4^–/–^ mice show attenuated neuronal cell death after MPTP induction, but CD8^–/–^ mice do not (Brochard et al., 2009). Reconstitution of SCID mice with WT CD4^+^ T cells abolishes this attenuated neuronal death (Gonzalez et al., 2013). CD4^+^ T cell-mediated neurodegeneration requires the expression of FasL but not IFN-γ, suggesting cytotoxic mechanisms of cell death (Brochard et al., 2009).

Besides Th1 cells, Th17 cells and the cytokine IL-17 have also been associated with PD. Immunization of animals with nitrated α-synuclein induces an adaptive immune response in the MPTP model. The adoptive transfer of immune cells from immunized mice into recipients prior to MPTP exacerbated neuroinflammation and neurodegeneration, a mainly Th17-mediated effect (Reynolds et al., 2010). CD4^+^ T cells isolated from immunized mice displayed a shift toward a Th17/Th1 phenotype, through the production of pro-inflammatory and neurotoxic cytokines such as IL-17, TNF-α, and IFN-γ (Reynolds et al., 2010). Adoptive transfer of *ex vivo* polarized T cells from immunized mice similarly showed that Th17 cells exacerbate neuronal loss, while Th1 cells show only a slight increase in neurodegeneration (Reynolds et al., 2010). Further supporting the role of IL-17 in PD pathogenesis, autologous co-cultures of iPSC-derived midbrain neurons and activated T cells from PD patients showed increased neuronal cell death, associated with an increased production of T cell-derived IL-17 and upregulated IL-17 receptor on neurons (Sommer et al., 2019). This degenerative effect was abolished following the pre-treatment of neurons with IL-17 or IL-17 receptor neutralizing antibodies, suggesting a role for Th17 cells in PD neuronal death (Sommer et al., 2019). These studies highlight a disrupted balance of CD4^+^ T cell subsets, with a shift toward pro-inflammatory Th17/Th1 cells, causing the dopaminergic neurodegeneration seen in PD.

On the other hand, the regenerative capacity of the adaptive immune system in PD is predominantly mediated by Tregs. Mice immunized with copolymer 1 (Cop-1) to generate T cells which are non-encephalitic and MBP-specific are protected from neurodegeneration in response to neurotoxicity (Schori et al., 2001). Moreover, adoptive transfer of splenocytes from mice immunized with Cop-1 to MPTP recipient mice led to protection against dopaminergic neuronal cell death (Benner et al., 2004). Dopaminergic protection is mediated by the infiltration of donor T lymphocytes to the area of damage as the adoptive transfer of T cell-depleted splenocytes shows no protective effect (Benner et al., 2004). T lymphocyte secretion of IL-4 and IL-10 suppresses microglial activation and induces astrocytic production of glial cell-derived neurotrophic factor (GDNF), a factor contributing to neuroprotection (Benner et al., 2004). Adoptive transfer of activated Tregs following MPTP induction resulted in over 90% survival of dopaminergic neurons, whilst the adoptive transfer of effector T cells showed no effect (Reynolds et al., 2007). Treg-mediated neuroprotection was conferred by modulation of neuroinflammation, increased neurotrophic production and suppressed microglial responses to stimuli, including aggregated α-synuclein (Reynolds et al., 2007). This was further validated *in vitro*, where co-cultures of microglia activated by α-synuclein and T cell subsets reveal that Tregs modulate microglial production of reactive oxygen species and the activation of nuclear factor kappa B (NFKB) (Reynolds et al., 2009). In contrast, effector T cells aggravate microglial inflammation and neurotoxicity (Reynolds et al., 2009).

Granulocyte-macrophage colony-stimulating factor (GM-CSF) is also considered neuroprotective in both animal models of PD and in PD patients, with neuroprotection primarily mediated through elevated Treg levels (Kosloski et al., 2013; Gendelman et al., 2017; Schutt et al., 2018). Treatment of mice with GM-CSF prior to MPTP intoxication protected against dopaminergic neuronal loss, with increased proportions of Tregs and reduced microgliosis (Kosloski et al., 2013). Adoptive transfer of CD4^+^ T cells or Tregs from GM-CSF treated mice following MPTP intoxication protected dopaminergic neurons and attenuated microglial activation (Kosloski et al., 2013). Clinical trials using recombinant GM-CSF (sagramostim) have also shown increased levels of Tregs and improved motor function in PD patients (Gendelman et al., 2017). Although these studies highlight the protective role of Tregs in preventing dopaminergic neuronal damage, they do not assess the role of T cells in PD-related regeneration.

Similarly to AD, age is a major risk factor for PD, with older onset being associated with a more severe disease phenotype and disability status (Pringsheim et al., 2014). Aging results in greater motor dysfunction, dopaminergic disturbances and a reduction of α-synuclein and total tau in the CSF (Mehanna et al., 2014; Szewczyk-Krolikowski et al., 2014; Pagano et al., 2016). Although age does not influence the common pathological final stages of PD, it does impact disability milestones over the early and progressive phases (Kempster et al., 2010). The mechanistic impact on disease progression and severity remains to be elucidated, with speculation primarily given to how aging impacts cell numbers and signaling in patients. The number of Tregs increase with age, however, no difference was observed in total Treg numbers or functionality in healthy elderly controls and elderly PD patients (Rosenkranz et al., 2007). Neurotoxin exposure occurring in PD exacerbates the neuroinflammation and oxidative stress seen during normal aging, leading to downregulation of the Wnt/β-catenin signaling pathway, which is essential for dopaminergic neurogenesis (Marchetti et al., 2020). This suggests that immune alterations in aging patients could potentially contribute to age-associated PD pathology.

## Amyotrophic Lateral Sclerosis

Amyotrophic lateral sclerosis is a classical neurodegenerative disease of the CNS, mainly characterized by motor dysfunction including spasticity, muscle weakness and dysphagia (Hardiman et al., 2017). ALS causes degeneration of motor neurons, resulting in progressive muscle weakness and subsequent paralysis, ultimately ending in respiratory failure and death (Hardiman et al., 2017). Affecting both upper and lower motor neurons, ALS mainly leads to motor symptoms. However, a growing body of literature has recently shown that non-motor symptoms such as cognitive and behavioral deficits, similar to those observed in frontotemporal dementia (FTD), can be seen in ALS patients (Ferrari et al., 2011; Phukan et al., 2012; Hardiman et al., 2017). The exact pathophysiological mechanism of ALS is unknown, however, protein aggregation in motor neurons and surrounding oligodendrocytes represents a hallmark of the disease. TAR DNA-binding protein 43 (TDP-43) is the main component of these aggregates and is detected in most ALS patients (Neumann et al., 2006). The accumulation of other misfolded proteins has also been found in specific ALS subtypes, such as misfolded superoxide dismutase 1 (SOD1) and fused in sarcoma (FUS) proteins (Morgan and Orrell, 2016). Similar to other neurodegenerative diseases, aging is a major risk factor for ALS development, with peak incidence between 75 and 79 years of age (Alonso et al., 2009).

Although adaptive immunity is not the central pathogenic mechanism of ALS, T lymphocytes displaying pro-inflammatory features contribute to ALS progression and severity. Infiltrated T lymphocytes were found in the post-mortem spinal cord of ALS patients (Engelhardt et al., 1993). Studies evaluating immune cell profiles in the peripheral blood of healthy controls and ALS patients showed no differences in levels of total lymphocytes or CD3^+^ T lymphocytes, however, highlighted differences within specific T cell subsets (Saresella et al., 2013; Shin et al., 2013; Chen et al., 2014; Jin et al., 2020). ALS patients have significantly lower proportions of CD4^+^ T cells with unchanged or elevated CD8^+^ T cell proportions (Chen et al., 2014; Jin et al., 2020). Despite this decreased CD4:CD8 T cell ratio, only a higher CD4^+^ T cell percentage in ALS patients correlates with disease severity and progression (Shi et al., 2007; Chen et al., 2014). This correlation was associated with a shift toward a Th1/Th17 cell-mediated pro-inflammatory immune response (Saresella et al., 2013; Jin et al., 2020). Even though the percentages of anti-inflammatory Th2 cells and Tregs were decreased (Saresella et al., 2013; Jin et al., 2020), higher percentages of IL-13-producing CD4^+^ T cells, a Th2-related cytokine, were detected in ALS patients, correlating with disease severity (Shi et al., 2007). Adding to the pro-inflammatory profile of ALS patients, there were also decreased proportions of BDNF-producing CD8^+^ T cells, which promote neuronal survival (Saresella et al., 2013). Furthermore, a study evaluating peripheral blood mononuclear cells (PBMCs) of monozygotic twins, one which had ALS and the other healthy, showed that the ALS twin had a more pro-inflammatory profile than her sister, with only her PBMC supernatant proving toxic to rat cortical neurons (Lam et al., 2016). Interestingly, although the findings show the presence of memory T cells in both twins, effector T cells were only found in the ALS twin, suggesting a role for effector T cells in this pro-inflammatory neurotoxic response (Lam et al., 2016).

The SOD1^*G93A*^ mouse model of ALS is an important tool for elucidating the role of the adaptive immune system in ALS pathology. This transgenic mouse model expresses a human SOD1 transgene with a G93A mutation, resulting in an excess of SOD1 protein causing degeneration of motor neurons and paralysis (Acevedo-Arozena et al., 2011). This model has shown that CD8^+^ T cells are involved in motor neuron loss following protein aggregation (Nardo et al., 2018; Coque et al., 2019). During the symptomatic stage of ALS pathology, CD8^+^ T cells infiltrate the CNS and contribute to neurodegeneration, as ablation of CD8^+^ T cells reduces motor neuron loss (Coque et al., 2019). SOD1 mutant CD8^+^ T cells secrete IFN-γ, inducing MHC-I expression and provoking neurodegeneration through Fas and granzyme cytotoxic pathways (Coque et al., 2019). Infiltrating CD8^+^ T cells in the spinal cord of ALS mice display a restricted T cell receptor repertoire, suggesting a self-directed immune response contributing to the selective ablation of motor neurons during ALS (Coque et al., 2019). A reduction of MHC-I expression and a lack of mature CD8^+^ T cells in the SOD1^*G93A*^ mice protects cervical motor neurons (Nardo et al., 2018).

The number of B lymphocytes is not altered in ALS patients and post-mortem spinal cord tissue showed a greater infiltration of T lymphocytes but not B lymphocytes (Engelhardt et al., 1993). B lymphocytes do not significantly impact ALS pathogenesis, since B cell-deficient SOD1^*G93A*^ mice show similar motor dysfunctions and survival rates to controls, and SOD1^*G93A*^-derived B cells show a similar phenotype to WT-derived B cells (Naor et al., 2009). In line with these findings, there are no differences in the levels of immunoglobulins in the peripheral blood of ALS patients compared to healthy controls (Chen et al., 2014). However, a unique glycan (A2BG2) which was discovered on the Fc domain of IgG antibodies in ALS patients has been implicated in ALS pathology and progression (Edri-Brami et al., 2012, 2015). These antibodies identify antigens located at the surface of motor neurons at the end stage of the disease, progressing cytotoxic neurodegeneration (Edri-Brami et al., 2015). Although there was no difference in B lymphocyte levels in ALS patients, B cells appear to have a role in neuronal cytotoxicity and death, through the production of specific IgG antibodies.

Whilst studies have shown a more pro-inflammatory immune profile in ALS patients, with correlations to disease severity, CD4^+^ T lymphocytes have also proven to be neuroprotective and regenerative in ALS. ALS mice deficient in functional T cells have shown an accelerated disease progression, with reduced microglia reactivity and decreased levels of the neuroregenerative insulin-like growth factor (IGF-1) (Beers et al., 2008; Chiu et al., 2008). This neuroprotective role was shown to be specifically CD4^+^ T cell-mediated, as SOD1^*G93A*^ mice lacking functional CD4^+^ T cells show accelerated motor neuron deficiency, reduced gliosis, increased pro-inflammatory markers and a reduction of trophic factors and glial glutamate transporters (Beers et al., 2008). Reconstitution with CD4^+^ T cells prolonged survival, with the mediation of microglial neuroprotection leading to ALS attenuation (Beers et al., 2008). SOD1^*G93A*^ mice also show an altered immune profile, similar to ALS patients, with decreased lymphoid numbers and T cell dysfunction (Banerjee et al., 2008). In particular, they have reduced Tregs as the disease progresses (Beers et al., 2011). Adoptive transfer of activated Tregs or activated effector T cells to SOD1^*G93A*^ mice resulted in the delayed loss of motor function and enhanced survival, results not seen with the adoptive transfer of naïve T cells (Banerjee et al., 2008). Interestingly, only Tregs delayed the onset of neurological symptoms and only effector T cells delayed progression in the late phase of the disease (Banerjee et al., 2008); suggesting differential roles for T lymphocytes depending on disease stage. Passive transfer of Tregs from SOD1^*G93A*^ mice at an early disease stage to SOD1^*G93A*^ mice lacking functional lymphocytes resulted in prolonged survival, with increased IL-4 levels and microglia modulation (Beers et al., 2011). Moreover, inducing Treg expansion in the SOD1^*G93A*^ mouse model of ALS resulted in increased neuroprotection, repression of astrocytic and microglial reactivity and increased neurotrophic factors, slowing disease progression and prolonging survival (Sheean et al., 2018).

As mentioned previously, ALS patients have a pro-inflammatory profile with decreased Tregs (Saresella et al., 2013; Jin et al., 2020). Multiple studies have shown that Tregs decrease during disease progression, with lower numbers predicting a more progressive disease course and diminished survival (Beers et al., 2011; Henkel et al., 2013; Rashid Chehreh Bargh et al., 2018; Sheean et al., 2018). These findings suggest that Tregs provide protection or regenerative properties during early stages of disease but ultimately decrease in number or functionality, allowing for disease progression. Tregs isolated from ALS patients were dysfunctional, with less effective suppression of T cell proliferation compared to healthy controls and a greater Treg dysfunction in rapidly progressing ALS patients (Beers et al., 2017). *In vitro* expansion of isolated ALS Tregs recovered suppressive capabilities, indicating a potential therapeutic target for the autologous transplant of expanded Tregs in slowing ALS progression through neuroprotection or neuroregeneration (Beers et al., 2017).

## Multiple Sclerosis

Multiple sclerosis is an immune-mediated disease of the CNS, the hallmark of which is demyelination followed by neurodegeneration. Although not a classical neurodegenerative disease, MS is characterized by neuroinflammation, demyelination, apoptosis of oligodendrocytes, astrogliosis and finally, axonal loss (Lassmann, 2018). The immune system is intrinsically linked to the risk and development of MS, with both genetic and environmental risk factors related to immunity. Multipoint linkage screens of families with MS show significant linkage within the MHC region, highlighting the importance of antigen presentation and the adaptive immune system in MS genetic risk (Sawcer et al., 2005). This was further supported by the discovery of MS risk alleles within the MHC region relating to T cell differentiation in genome-wide association studies (Sawcer et al., 2011; Alcina et al., 2012). Although genetic risk involves adaptive immunity, familial studies highlight a modest role for genetics in MS risk, with concordance rates for monozygotic twins around 15–25% (Ramagopalan et al., 2008; Westerlind et al., 2014). Disease-modifying therapies (DMT) prevent disease progression through different mechanisms of immunomodulation. As most DMTs either deplete T and B lymphocytes and monocytes (Coles et al., 2008; Giovannoni et al., 2010; Hauser et al., 2017) or prevent lymphocyte entrance into the CNS (Polman et al., 2006; Kappos et al., 2010), this identifies a key role for the adaptive immune system in the pathogenesis and progression of the disease, at least in the early stages.

Histological examination of brain biopsies from MS patients has indicated a role for the adaptive immune system, particularly T lymphocytes, in demyelination and neurodegeneration. Demyelinating lesions show an infiltration of peripheral T lymphocytes, with both CD4^+^ T cells and CD8^+^ T cells found at the edge of demyelinating lesions and less immune infiltration in chronic inactive lesions (Traugott et al., 1983). The T cell-mediated pathogenic mechanism has been thought to be a Th1 response, due to the expression of IFN-γ within lesions and the association of macrophages as the effector cells of Th1 immunity and demyelination (Traugott and Lebon, 1988). Although studies indicate that immunopathology in MS is T cell-driven, it is also hypothesized that immune infiltration is secondary to CNS insult. Histopathological analysis of newly forming lesions from MS patients describes minimal myelin loss, dysfunction and apoptosis of oligodendrocytes, reactive astrocytes and activated microglia as early features of lesion formation (Rodriguez et al., 1993; Barnett and Prineas, 2004). Infiltrating immune cells were a feature of lesion progression and were found in close proximity to deteriorating myelin sheaths (Rodriguez et al., 1993). Classification of active demyelinating lesions showed the heterogeneity and complexity of the pathogenesis of MS. Although lesions showed common pathology, different demyelinating mechanisms were observed between patients (Lucchinetti et al., 2000). These findings suggest that both hypotheses for the pathogenesis of MS may be correct depending on the patient in question.

The pathogenic immune-mediated mechanisms of demyelination and neurodegeneration have largely been hypothesized using the animal model, experimental autoimmune encephalomyelitis (EAE). EAE is induced through immunization with myelin peptides, CNS tissues, or through the adoptive transfer of myelin-specific T cells, showing the key features of MS pathology. Although this model is not an exact representation of MS, it has allowed for the study and validation of DMT’s (Constantinescu et al., 2011). The integral role of T lymphocytes in the induction of EAE has been highlighted, as mice depleted of T cells do not develop EAE or produce MBP-specific autoantibodies, whereas mice depleted of B cells develop normal EAE in the absence of MBP-specific autoantibodies (Ortiz-ortiz and Weigle, 1976; Hjelmström et al., 1998). Further supporting this finding, the adoptive transfer of MBP-specific clonal T cells from rats immunized with MBP induced paralysis, meningeal inflammation and demyelinated lesions in recipients (Zamvil et al., 1985). This effect is lost when cells were first depleted of CD4^+^ T cells, and remained when depleted of CD8^+^ T cells (Pettinelli and McFarlin, 1981). During active demyelination and recovery in EAE, inflammatory aggregates within the perivascular space predominantly contain CD4^+^ T cells but also contain CD8^+^ T cells and B cells (Sriram et al., 1982). Although EAE is induced by CD4^+^ T cells, B lymphocytes are also implicated in demyelination and neurodegeneration, as shown by IgG antibody deposits present on degenerating myelin sheaths in both MS patient lesions and a primate model of EAE (Raine et al., 1999).

Experimental autoimmune encephalomyelitis induction has been further narrowed down to CD4^+^ T cell subsets. MOG-specific T cells polarized to Th1, Th17, and Th9 all induced EAE after adoptive transfer to recipients but Th2 polarized cells did not (Jager et al., 2009). Th1 and Th17 cells showed classical inflammatory infiltration and demyelinating lesions, however, when Th17 cells were cultured with IL-23, lymphoid follicle-like structures were observed (Jager et al., 2009). Th9 cells similarly induced extensive demyelination within both the CNS and PNS (Jager et al., 2009). Adoptive transfer of Th1 cells induces ‘classical’ EAE with paralysis developing from tail to head while the adoptive transfer of Th17 cells induces ‘atypical’ EAE with ataxia, unbalanced gait and rotary defects progressing to paralysis (Domingues et al., 2010). Adoptive transfer of both, however, induces a more severe disease (Domingues et al., 2010). The differences seen in EAE may represent the heterogeneity seen in MS patients and may be indicative of different disease mechanisms in patients.

The role of the adaptive immune system in the pathogenesis of MS is well established, however, the role of the adaptive immune system in regeneration is more novel. Regeneration in MS is centered on the differentiation of oligodendrocyte progenitor cells (OPC) into oligodendrocytes, which replace myelin sheaths to prevent axon degeneration in a process known as remyelination (Smith et al., 1979; Franklin and Ffrench-Constant, 2017). T lymphocytes have been shown to have regenerative effects in the CNS, with Rag1^–/–^ mice, lacking mature lymphocytes, displaying reduced remyelination of lysolecithin-induced demyelinating lesions compared to WT controls (Bieber et al., 2003). This impaired remyelination was also observed in mice deficient or depleted of either CD4^+^ or CD8^+^ T cells, highlighting the important role for T cells in myelin regeneration (Bieber et al., 2003). Tregs have been shown to be key in this process, promoting OPC differentiation and efficient remyelination (Dombrowski et al., 2017). Conversely, not all CD4^+^ T cell subsets are beneficial, as Th17 cells proved detrimental for remyelination in a model of cuprizone-mediated demyelination (Baxi et al., 2015).

Similar to other neurodegenerative diseases, aging is an important risk factor for MS and is implicated in disease onset, progression and evolution. However, the role of aging in disease susceptibility and severity is controversial in EAE models. Some studies suggest that aging decreases disease susceptibility (Djikić et al., 2015), whilst others report an increased susceptibility or severity with aging (Matejuk et al., 2005; Seo et al., 2015; Stojić-Vukanić et al., 2015). Further studies have also indicated that with aging, mice develop chronic EAE that is more similar to the symptoms observed in progressive MS (Ludowyk et al., 1993; Peferoen et al., 2016). Research performed with MS patients indicated that disability milestones are mainly determined by patient age and age at disease onset, indicating age-related mechanisms of disease development (Confavreux and Vukusic, 2006). The probability of incomplete recovery and relapse severity also increases with age, leading to the accumulation of disability (Cossburn et al., 2012; Kalincik et al., 2014). This is mainly mediated by age-associated impaired remyelination, as shown in lysolecithin-induced demyelinating lesions in juvenile, young and old rats. Old rats (>12 months) showed decreased remyelination of axons and thinner myelin sheaths for those that did remyelinate, compared to their young and juvenile counterparts (Gilson and Blakemore, 1993). The lesions of older animals also contained more myelin debris, indicating that remyelination failure may be due to a delayed damage response from astrocytes and macrophages, with deficient myelin debris clearance (Kotter et al., 2006; Linehan et al., 2014; Natrajan et al., 2015; Cantuti-Castelvetri et al., 2018). This aging-related failure may also be mediated by the reduced or slower recruitment of OPCs (Gilson and Blakemore, 1993), and the delayed differentiation of OPCs into oligodendrocytes (Sim et al., 2002). Recently, it has also been shown that aging impairs the response of OPCs to pro-differentiation cues such as the thyroid hormone (Neumann et al., 2019), suggesting that enhancement of OPC differentiation by the adaptive immune system may be altered with aging.

## Stroke

Globally, stroke represents the second highest cause of death and commonly results in disability (Campbell et al., 2019). There are two classifications of stroke: ischaemic and haemorrhagic stroke. The majority of cases are ischaemic, which are caused by arterial occlusion, often by emboli originating in the heart or large arteries, but also by local thrombosis, vasculitis or arterial dissection (Campbell et al., 2019). Although stroke is not a classical neurodegenerative disease, tissue ischaemia causes neuronal death and is associated with a rapid local innate immune response, upregulation of pro-inflammatory cytokines, BBB breakdown and infiltration of peripheral immune cells (Kamel and Iadecola, 2012).

Tissue ischaemia promotes activation of adaptive immune networks with increased infiltration of antigen-presenting cells in the CNS (Felger et al., 2010), expansion of CNS antigen-specific T cells (Jin et al., 2018) and increased immunoglobulin synthesis in the CSF (Pruss et al., 2012). There is mounting evidence that the adaptive immune system contributes to the pathogenesis and evolution of acute ischaemic stroke. Patients that die following a stroke (<24 h) show an increased infiltration of neutrophils, B lymphocytes, CD3^+^ T cells and CD4^+^ T cells in the infarcted area (Gelderblom et al., 2012; Clarkson et al., 2014; Doyle et al., 2015). This is supported in experimental stroke animal models, with higher T lymphocyte numbers at the edge of the infarcted tissue early after induction (Schroeter et al., 1994). Moreover, IL-17A^+^ T lymphocytes were detected in the post-mortem tissue of patients that died shortly after their stroke (Gelderblom et al., 2012) and IL-21-producing CD4^+^ T cells, potentially Th17, Tfh, or Th9 cells, were found surrounding the infarcted tissue area in post-mortem tissue of patients with acute stroke (Clarkson et al., 2014).

Much of the evidence linking the adaptive immune system to stroke is from the common animal model used to study its pathogenesis: the middle cerebral artery occlusion model (MCAO). In MCAO, a surgical filament is inserted into the external carotid artery to occlude the origin of the middle cerebral artery, resulting in blood flow cessation and brain infarction in the striatum (Chiang et al., 2011). T cell involvement in the progression of neurodegeneration following experimental stroke induction has been studied by the depletion of adaptive immune cell subsets. SCID mice, deficient in mature lymphocytes, showed a reduction in total infarct size after MCAO with little effect on the infarct core, suggesting that lymphocytes promote the progression of ischaemic neurodegeneration following initial vascular insult (Hurn et al., 2007). Similar results were also observed in lymphocyte-deficient Rag1^–/–^ mice (Kleinschnitz et al., 2010). The susceptibility of these mice to MCAO was restored with the adoptive transfer of CD3^+^ T cells but not with the transfer of B cells, highlighting the role of T lymphocytes in progressive ischaemic neurodegeneration (Kleinschnitz et al., 2010). Despite not having a direct role in stroke pathogenesis, B lymphocyte responses have been shown to cause a delayed cognitive impairment in stroke mouse models, without impacting infarct size (Doyle et al., 2015).

Depletion of CD8^+^ T cells has been shown to reduce infarct size and behavioral deficits following MCAO, whilst the adoptive transfer of CD8^+^ T cells in Rag1^–/–^ mice increased infarct size (Mracsko et al., 2014). CD4^+^ T cells also contribute to neurodegeneration following stroke, as antibody-mediated depletion of either CD4^+^ or CD8^+^ T lymphocytes decreased infarct size, outlining a role for both subsets (Liesz et al., 2011). This study demonstrated the role of both humoral and cytotoxic T cell responses in promoting ischaemic infarction, whilst the inhibition of lymphocyte trafficking to the CNS resulted in a protective effect (Shichita et al., 2009; Liesz et al., 2011). The use of different knockout transgenic mouse models, has shown that this early pathogenic role of T lymphocytes can occur independently of classical adaptive immune mechanisms, such as antigen-recognition or TCR co-stimulation (Kleinschnitz et al., 2010). Although these mechanisms do not seem to be required for the initial stages of T cell-mediated ischaemic brain injury, the role of T lymphocytes in the progression of neurodegeneration does appear to be antigen-dependent (Kleinschnitz et al., 2010; Mracsko et al., 2014).

The study of T cell-derived cytokines provides an insight into the degenerative effects of the adaptive immune system following a stroke. Depletion of CD4^+^ T cells 3 days post-stroke induction improved behavioral outcomes without affecting infarct size, through the reduction of inflammatory cytokines such as IFN-γ and IFN-γ-inducible protein (IP-10) (Harris et al., 2020). In addition, IL-17-producing γδ T cells, have been found at the infarct edge and have been implicated in the delayed phase of infarction, with IL-17-deficient mice showing reduced neuronal death and improved neurological outcome (Shichita et al., 2009). Furthermore, neutralization of IL-17A following MCAO improved neurological outcome and reduced infarct size (Gelderblom et al., 2012). The blockade of IL-21, produced by infiltrating CD4^+^ T cells, either before or after MCAO induction showed reduced infarct size and increased locomotor function (Clarkson et al., 2014). Despite the knowledge gained from the use of experimental stroke in rodents, there has so far been little success in translating immunosuppressive therapies into the clinic, with natalizumab, a drug preventing lymphocyte infiltration into the CNS, having no effect in stroke patients during phase II clinical trials (Elkins et al., 2017).

There is also evidence that systemic inflammation during stroke can lead to long-term autoimmunity with further neurodegeneration. BBB disruption during ischaemic tissue injury promotes secretion of pro-inflammatory cytokines and sensitisation to CNS antigens, associated with poorer neurological outcomes (Javidi and Magnus, 2019). The long-term autoimmune response is exacerbated in the context of systemic inflammation, as mice treated with lipopolysaccharides (LPS) are more likely to be sensitized to MBP after MCAO and show greater neurological deficits when compared to non-LPS treated mice (Becker et al., 2005). In addition, T cells isolated from patients following acute ischaemic stroke were more likely to react to CNS myelin antigens than those of control patients with other neurological diseases (Wang et al., 1992). Following stroke there is an increase in antibody titres recognizing CNS self-antigens, indicating a B cell response following ischaemic brain injury (Dambinova et al., 2003). The relationship between poorer neurological outcomes and systemic inflammation during stroke has also been highlighted, as patients who developed an infection following their stroke were more likely to have MBP and GFAP-specific T cells 3 months post-stroke; associated to a Th1 response (Becker et al., 2011). Establishing immune-tolerance can mitigate some of the adverse effects of the adaptive immune response, with the generation of oral tolerance to MBP in rats reducing infarct size following subsequent MCAO (Becker et al., 1997). In line with this finding, it has also been shown that IL-10-producing CD4^+^ T cells are important for mediating mucosal tolerance to CNS antigens and can limit damage after an ischaemic stroke (Frenkel et al., 2005).

The adaptive immune system is, however, also partly involved in neuroprotection and neuroregeneration following ischaemic tissue injury, due to the infiltration of Tregs; though there is debate about the timing of infiltration and the nature of these Tregs in ischaemic stroke (Ito et al., 2019; Javidi and Magnus, 2019). Tregs that accumulate post-damage are molecularly distinct from those in other tissues and regulate neurotoxic astrogliosis, promoting neurological recovery during the chronic stroke phase (Ito et al., 2019). The beneficial role of T lymphocytes has also been demonstrated by treatment with fingolimod to sequester T cells in lymph nodes 6–13 days following stroke, which delayed neurological recovery (Ito et al., 2019). Depletion of Tregs a week following stroke similarly showed a poorer neurological outcome without affecting infarct size (Ito et al., 2019), whereas depletion of Tregs 48 h before MCAO resulted in the increased activation of microglia and T cells, sources of the pro-inflammatory cytokines TNF-α and IFN-γ (Liesz et al., 2009). Treg depletion led to increased chronic ischaemic brain damage and a worsened neurological outcome with increased infarct size after 7 days (Liesz et al., 2009). Treatment with IL-10, on the other hand, reduced TNF-α and IFN-γ cytokine overexpression and prevented secondary infarct progression (Liesz et al., 2009). Additionally, administration of CD34^+^ immune progenitor cells, which can differentiate into both innate and adaptive immune cells, promoted revascularisation and enhanced neurogenesis after MCAO induction (Taguchi et al., 2004), being now further evaluated in clinical trials (Sargento-Freitas et al., 2018). Although these studies highlight the ability of adaptive immune cells to reduce neurodegeneration, it can be difficult to establish whether these effects are through neuroprotection or by promoting neuroregeneration and plasticity.

Aging is also an important risk factor for stroke, with the vast majority of cases occurring in people over the age of 75 (Engstad et al., 2012). Elderly patients have a greater risk of fatality, are more likely to require longer hospitalization and are less likely to return home following stroke (Saposnik et al., 2008). Although it has been shown that older stroke patients make a less complete neurological recovery than their younger counterparts, this data is complicated due to other cofounding morbidities in elderly patients. Behavioral recovery has been shown to be delayed in older mice, despite infarct size being lower than younger controls; potentially caused by an enhanced innate immune response and increased reactive gliosis (Manwani et al., 2011). The immunological response to stroke is also exacerbated in older animals (Manwani et al., 2013), with bone marrow transplants from young mice improving stroke outcomes (Ritzel et al., 2018). The numbers of CD8^+^ T cells in the CNS are increased in aged animals, regardless of tissue injury (Ritzel et al., 2016). Following the induction of experimental stroke, these resident CD8^+^ T cells exacerbate ischaemic brain injury by potentiating further leukocyte recruitment from the periphery and amplifying pro-inflammatory cytokines in aged animals (Ritzel et al., 2016). Older animals with chronic systemic infection similarly show increased immune cell recruitment, upregulation of pro-inflammatory cytokines and increased infarct size compared to young controls with systemic infection (Dhungana et al., 2013). This suggests that the inflammatory response of elderly patients, which tends to be dysregulated, is an important parameter in the severity of ischaemic tissue injury.

## Traumatic CNS Injury

Traumatic CNS injury is a broad term encompassing damage to the brain (TBI) or spinal cord (SCI) commonly caused by a sudden, external impact. The primary insult is a determinant of the extent of neurodegeneration and outcome for patients, however, neuronal loss is often the result of secondary neurodegeneration. In SCI, damage is often caused by displaced surrounding structures causing bruising or tearing of the spinal cord, leading to either complete or incomplete classifications of SCI (Alizadeh et al., 2019). Secondary neurodegeneration often occurs as a result of the altered tissue environment following damage, with ischaemia, oxidative stress, glial activation, matrix remodeling and neuroinflammation all contributing to progressive damage (Alizadeh et al., 2019). TBI, on the other hand, comprises a diverse range of pathologies, including focal damage due to bruising, laceration or traumatic hemorrhage, as well as more diffuse effects resulting from acceleration/deceleration injuries (Werner and Engelhard, 2007). Secondary neurodegeneration is a common feature, with ischaemia, oedema formation, intracranial hypertension and neuroinflammation causing neuronal death (Werner and Engelhard, 2007). TBI is also a risk factor for chronic neurodegeneration, particularly chronic traumatic encephalopathy (McKee et al., 2009). Although it is necessary to evaluate both the brain and spinal cord in the inflammatory response to traumatic injury, many researchers focus on SCI due to the greater inflammatory response seen in SCI animal models (Schnell et al., 1999).

There is little evidence that the adaptive immune system exacerbates pathology acutely following TBI, however, there is evidence of it contributing to TBI progression. TBI patients show progressive degeneration of the white matter and persistent inflammation many years after the initial injury (Johnson et al., 2013). Both CD4^+^ and CD8^+^ T cells infiltrate the CNS following contusion TBI (Holmin et al., 1995). Inflammation persists several months after focal TBI, with upregulation of MHC-II, phagocytes and pro-inflammatory cytokines IL-1β and TNF-α (Holmin and Mathiesen, 1999). Brain biopsies from patients undertaking surgeries for brain contusions showed a limited inflammatory response <24 h post-injury, however, by 3–5 days there was a substantial immune infiltrate of reactive microglia, macrophages, polymorphonuclear cells, and both CD4^+^ and CD8^+^ T lymphocytes (Holmin et al., 1998). Neuroinflammation is known to be involved in progressive brain damage with pre-clinical data for several immunosuppressive therapies showing promising results. However, this has not translated to give clear benefits in clinical trials, with some drugs worsening neurological outcome (Bergold, 2016; Russo and McGavern, 2016).

Progressive neurodegeneration following TBI is mediated by adaptive immunity. Inhibition of antigen processing and presentation impairs immune cell infiltration to the CNS, resulting in less neurodegeneration and a smaller lesion size following fluid percussion TBI (Tobin et al., 2014). Other models argue against a role for the adaptive immune system in neurodegeneration after TBI (Weckbach et al., 2012; Mencl et al., 2014). Rag1^–/–^ mice, lacking functional lymphocytes, show a similar extent of neurological injury compared to controls following closed head injury (Weckbach et al., 2012). Additionally, fingolimod treatment to sequester T cells and prevent CNS infiltration had no impact of lesion size or functional outcome following focal cortical cryo-lesion TBI (Mencl et al., 2014). This study also observed a reduced number of neutrophils and activated microglia/macrophages in lesions, highlighting an adaptive immune role in sustaining neuroinflammation (Mencl et al., 2014). These divergent results may be due to the pathologically diverse lesions induced through a wide range of animal models, representing the spectrum of TBI. Autoimmunity is also a feature of adaptive immunity following TBI, with myelin-reactive circulating T cells in patients and CNS autoantibodies developing upon contusion injury in rats (Cox et al., 2006; Rudehill et al., 2006).

Similarly, SCI patients show an altered immune profile with suppressed and defective immune responses, known as CNS injury-induced immunodepression (CIDS), a phenomenon which also occurs in TBI patients (Meisel et al., 2005; Zhang Y. et al., 2013; Gucluler et al., 2017). SCI patients have a higher susceptibility to infections due to a decline in both innate and adaptive immune responses (Gucluler et al., 2017). SCI insult induces a potent inflammatory response with subsequent anti-inflammatory mechanisms systemically and within the damaged area, leading to suppression of immunity. Immune cells’ functionality continues to evolve as the injury progresses from primary insult to secondary neurodegeneration (Schwab et al., 2014). For example, in a transection mouse model of SCI, neutrophils and T lymphocytes persist at the acute phase of injury, with a 50% reduction after 1–3 days, whilst microglia and macrophages persist into the chronic phase, falling by 50% only after 55 days (Pruss et al., 2011). Despite this early reduction, 10% of T lymphocytes persisted after 4 weeks, adding to the sustained neuroinflammation seen following acute SCI (Pruss et al., 2011). Other studies have highlighted different dynamics of lymphocyte infiltration after injury, although studies agree on the persistence of T lymphocytes several weeks after injury (Popovich et al., 1997; Schnell et al., 1997; Sroga et al., 2003). A similar response was seen in post-mortem spinal cord tissue from SCI patients with different survival times of up to 1 year post-trauma (Fleming et al., 2006). The innate immune response occurred quickly following SCI and T lymphocytes increased in number at 1–6 months post-injury, predominantly CD8^+^ T cells (Fleming et al., 2006).

Similar to other CNS neurodegenerative diseases, BBB disruption following injury can lead to autoimmunity. This effect is seen in patients and mouse models of SCI, with detection of MBP-specific T cells and the activation of MBP-reactive T lymphocytes from SCI donors promoting neuroinflammation and inducing transient paralysis in donor mice (Popovich et al., 1996). In patients with SCI, the frequency of MBP-reactive T cells reach levels similar to that seen in MS patients (Kil et al., 1999). These MBP-reactive T lymphocytes have been suggested to contribute to neurodegeneration and impede recovery following SCI, with SCI induced in 2D2 mice, with >95% of all CD4^+^ T cells reactive to MBP, showing exacerbated neuropathology (Jones et al., 2002). Likewise, induction of contusion SCI in mice leads to a dysregulation of B lymphocytes, with increased numbers and production of neurotoxic CNS autoantibodies (Ankeny et al., 2006).

To address the functional role of T lymphocyte infiltrates in SCI, mice lacking components of the adaptive immune system were utilized. Researchers showed that lymphocytes contribute to neurodegeneration and worsened functional recovery following crush SCI in SCID mice (Luo et al., 2019). Post-damage, animals showed a reduced inflammatory response, reduced immune function-related gene expression, increased neural transmission-related gene expression, smaller lesion size and an improved recovery of motor function after injury (Luo et al., 2019). Similar results were obtained in Rag2^–/–^ mice and athymic nude rats, which lack mature lymphocytes or T cells, respectively, with decreased secondary neurodegeneration, increased regeneration and improved functional recovery (Potas et al., 2006; Wu et al., 2012). Dorsal hemisection SCI in mice leads to an elevated expression of a T cell chemoattractant, CXCL10 (Gonzalez et al., 2003). Its neutralization 1 day post-injury led to reduced T cell accumulation in the CNS, with decreased lesion size and reduced behavioral deficits, supporting the detrimental role of T cells in SCI (Gonzalez et al., 2003).

B lymphocytes and immunoglobulins have also been associated with progressive neuronal degeneration following traumatic CNS injury, with large antibody deposits detected at sites of axon degeneration and demyelination following contusion SCI (Ankeny et al., 2009). Depletion of B cells after compression SCI caused immunomodulatory effects with decreased neuronal death and delayed motor dysfunction (Casili et al., 2016). BCKO mice, lacking B lymphocytes, also showed reduced lesion pathology and improved locomotor recovery associated with lack of immunoglobulins in the CSF of injured mice (Ankeny et al., 2009). Previously mentioned expression of new epitopes expressed following SCI are detected by clonally specific IgM antibodies, resulting in complement activation and worse pathology (Narang et al., 2017). Rag1^–/–^ mice, which are less susceptible to neurodegeneration following injury, administered with specific IgM antibodies, showed exacerbated pathology and a worsened functional outcome (Narang et al., 2017). These findings together support a role for both, B and T lymphocytes in the development and progression of traumatic CNS injury.

Despite the role in progressing secondary neurodegeneration and neuroinflammation following traumatic injury to the CNS, adaptive immunity and specifically autoimmunity have also shown neuroprotective and regenerative properties. Co-culture of neurons and immune cell subtypes, highlight the regenerative capacity of CD4^+^ T cells which augment neurite outgrowth (Pool et al., 2012). CD8^+^ T cells inhibited this process, showing the heterogeneity of lymphocytes in CNS repair (Pool et al., 2012). Adoptive transfer of *ex vivo* polarized Th1 cells following SCI in WT mice enhanced recovery of locomotor function, promoting regeneration of the corticospinal tract, serotonergic fibers and myelin (Ishii et al., 2012). This regenerative effect may have been mediated by the Th1 production of NT3 or IL-10, as IL-10 neutralization attenuated the positive effects while adoptive transfer of either Th2 or Th17 cells did not show the same effects (Ishii et al., 2012). Another study has shown that adoptive transfer of CD4^+^ T cells into IL-4-deficient mice after CNS injury promotes neuronal survival and regeneration of injured neurons, an effect not seen with the adoptive transfer of IL-4-deficient CD4^+^ T cells (Walsh et al., 2015). The IL-4-producing T cell-mediated effect, indicative of Th2 cells, was shown to be MHC-II-independent and MyD88-dependent (Walsh et al., 2015). In line with these studies, vaccination with Cop-1 following closed head TBI caused reduced neuronal loss and promoted recovery, thought to be mediated by increased immune repair mechanisms by Cop-1 treatment (Kipnis et al., 2003).

Autoimmunity induced following traumatic CNS injury is observed in both the brain and spinal cord, however, there is debate on whether this is detrimental or beneficial in CNS repair. Active immunization with MBP or transfer of MBP-specific T lymphocytes following contusion SCI promoted neuronal regeneration and improved motor function (Hauben et al., 2000). Similar results were seen in dorsal hemisection injury, with myelin or spinal cord homogenate immunization promoting long-distance regeneration of axons, corticospinal tract sprouting and improved locomotor activity (Huang et al., 1999; Sicotte et al., 2003). Immunized mice can develop autoantibodies against endogenous inhibitors of neurite growth found in the adult CNS, which can in turn promote neurite growth *in vitro* (Huang et al., 1999). These studies highlight the neuroprotective and neuroregenerative roles of T cell-mediated immune activity following CNS injury.

As traumatic CNS injury is dependent on the external trauma, it is the only neurodegenerative disease discussed here for which aging is not a risk factor. However, older patients with TBI show a less complete recovery at 1 year post-injury, with more severe consequences following trauma (Rothweiler et al., 1998); suggesting that age-associated regeneration failure will also influence recovery in traumatic CNS injury.

## Aging – a Key Factor Modulating CNS and Immune System Interactions

As discussed above, aging is a major risk factor in neurodegenerative diseases, with aged individuals often presenting with more severe forms of disease or incomplete recovery after damage. Studies have discussed the impact or mechanisms of aging-related pathogenesis in specific neurodegenerative diseases, however, research into the normal physiological changes that occur during aging can facilitate our understanding of disease development or progression with age. Like all biological entities, the CNS and the adaptive immune system functionally decline with healthy aging, with brain atrophy and increased levels of neuroinflammation. Although the CNS was previously considered an ‘immune-privileged site,’ with immune infiltration only occurring after BBB disruption caused by damage or aging, studies have shown that the adaptive immune system is present and beneficial in the healthy CNS (Ellwardt et al., 2016). CD4^+^ and CD8^+^ T lymphocytes are present in the healthy CNS and are required for immune-surveillance and protection against neurotropic viruses (Kivisäkk et al., 2003; Smolders et al., 2013, 2018). Infiltrating T lymphocytes regulate the integrity and homeostasis of the CNS, inducing hippocampal neurogenesis and improving cognitive function in healthy rats (Ziv et al., 2006). As cognitive function declines during aging, this may suggest a dysfunctional CNS-resident T cell population.

Functional decline of the aging adaptive immune system is termed immunosenescence and is associated with greater susceptibility to infections. Although cellular senescence occurs, aging also induces dysfunctional systemic inflammation (inflammaging) through immune cell alterations. This dysfunction may be due to heterogeneous epigenetic modifications identified in PBMC’s of older individuals, showing a clear aging signature (Ucar et al., 2017; Cheung et al., 2018). During aging, there is an increased number of inflammatory cells in the CNS, contributing to higher levels of neuroinflammation (Stichel and Luebbert, 2007; Dulken et al., 2019). Increased infiltration may result from aging-related alterations of the BBB and its transport mechanisms, leading to increased BBB permeability (Shah and Mooradian, 1997; Bake et al., 2009; Blau et al., 2012; Lee et al., 2012). Alongside increased permeability, the aged rat brain also showed decreased perfusion, promoting microglial activation and infiltration of macrophages which express lymphocyte and monocyte chemo-attractants, IP-10 and MCP-1 (Blau et al., 2012). Compounding this reduction in vascular perfusion, the aging brain is also less efficiently drained by meningeal lymphatic vessels, associated with impaired clearance of macromolecules and dysregulated inflammation-associated gene expression in lymphatic endothelial cells (Da Mesquita et al., 2018). Together, these mechanisms could contribute to the greater infiltration of lymphocytes into the aging brain. Greater infiltration was confirmed by single-cell analysis of young and old mice showing an increased T cell population, predominantly CD8^+^ T cells, within neurogenic niches in aged animals (Dulken et al., 2019). This age-associated increase in CD8^+^ T cells leads to increased IFN-γ signaling and a subsequent decrease in neurogenesis (Dulken et al., 2019). A similar study showed an aging-related increase in number and accumulation of CD3^+^ T cells and dendritic cells in the brain parenchyma from 12 months onward, whilst B lymphocytes were not detected at any age (Stichel and Luebbert, 2007). Aged mice display an increase in numbers of naïve and memory CD4^+^ T cells which recognize foreign pMHC-II, however, decreased numbers were detected in the brain and draining lymph nodes of aged mice following neurotropic virus infection (Deshpande et al., 2018). This highlights the dysfunctional immune response in aged animals, with increased responding cell numbers not translating into a greater clearance of infection (Deshpande et al., 2018).

The aging-related deterioration of T cell homeostasis has been linked to the age-dependent involution of the thymus (Linton and Dorshkind, 2004). This chronic inflammation leads to an increased rate of brain CNS aging which in turn initiates systemic aging, highlighting the co-dependency of the two systems (Coder et al., 2017). This co-dependency is supported by the greater response of microglia and astrocytes in the aged brain to pro-inflammatory cytokines such as IFN-γ and TNF-α (Xu et al., 2010), which also shows an age-related increase in intercellular adhesion molecule (ICAM)-1 expression on astrocytes and microglia, promoting further T cell recruitment to the CNS (Xu et al., 2010). As a result, increased CD4^+^ and CD8^+^ T cells are observed in the brain parenchyma and choroid plexus during aging (Xu et al., 2010). Thymus involution and aging results in a decline of naïve T cells and peripheral B cells with an increase in the number of terminally differentiated T cells (Pulko et al., 2016; Cao and Zheng, 2018). Thymic involution also causes autoreactive T cell clones to be released into the periphery, as they are no longer depleted by the thymus, increasing susceptibility to autoimmunity and contributing to chronic inflammation (Coder et al., 2015). Aged naïve CD8^+^ T cells show defective expansion and differentiation following bacterial infection, with increased apoptosis of effector cells (Smithey et al., 2011). A large proportion of memory CD8^+^ T cells in aged mice develop without antigen stimulation and are the result of a switch from naïve T cells to a memory cell phenotype (Chiu et al., 2013; Moskowitz et al., 2017). These high-avidity memory-like virtual T cells are important compensatory cells during aging as aged naïve T cells have a less diverse repertoire of T cell receptors (Rudd et al., 2011).

Diminished numbers of circulating B lymphocytes during aging are associated with an altered and less diverse antibody repertoire (Gibson et al., 2009; Tabibian-Keissar et al., 2016; Riley et al., 2017; Nikolich-Zugich, 2018). Although numbers are decreased, the proportion of age-associated B cells (ABCs) increases (Hao et al., 2011; Riley et al., 2017). ABCs are pro-inflammatory cells which inhibit normal B cell differentiation and have a distinct antibody repertoire that is more reactive to self-antigens (Ratliff et al., 2013; Riley et al., 2017). There are alterations of B cell subsets in the peripheral blood of aged individuals, with increased proportions of late/exhausted B cells, similar to ABCs, expressing senescence-associated secretory phenotype (SASP) markers and activating NF-κB (Frasca et al., 2017; Frasca, 2018). Aging-associated disruption of B cell subsets results in a diminished response to exogenous antigens and vaccines with a greater susceptibility to infection (Pinti et al., 2016; Frasca et al., 2017). This diminished response may be mediated by a higher expression of TNF-α by ABCs, which in turn negatively affects other B cell subsets (Frasca et al., 2014). Aging-related B lymphocyte dysregulation is thought to occur over the age of 60, with genome-wide expression profiles of B cells from young and aged donors up to 60 years of age showing no differences in gene expression (Knight et al., 2016).

Analysis of aged individual’s blood showed increased pro-aging and immune factors, such as CCL11 and B2M, resulting in the development of an aging phenotype negatively affecting memory function and neurogenesis (Villeda et al., 2011; Smith et al., 2015). The main barrier between the circulation and the CNS, the choroid plexus, is home to CNS-specific CD4^+^ T cells. During aging and immunosenescence, these cells are shifted to a Th2 response, resulting in elevated levels of IL-4 and CCL11 which are detrimental for cognitive function (Baruch et al., 2013). Restoration of the cytokine balance between IL-4 and IFN-γ, with blockage of type I interferon signaling, can restore cognitive function, highlighting the disruption the aged immune system has on the CNS (Baruch et al., 2013, 2014).

**FIGURE 2:**
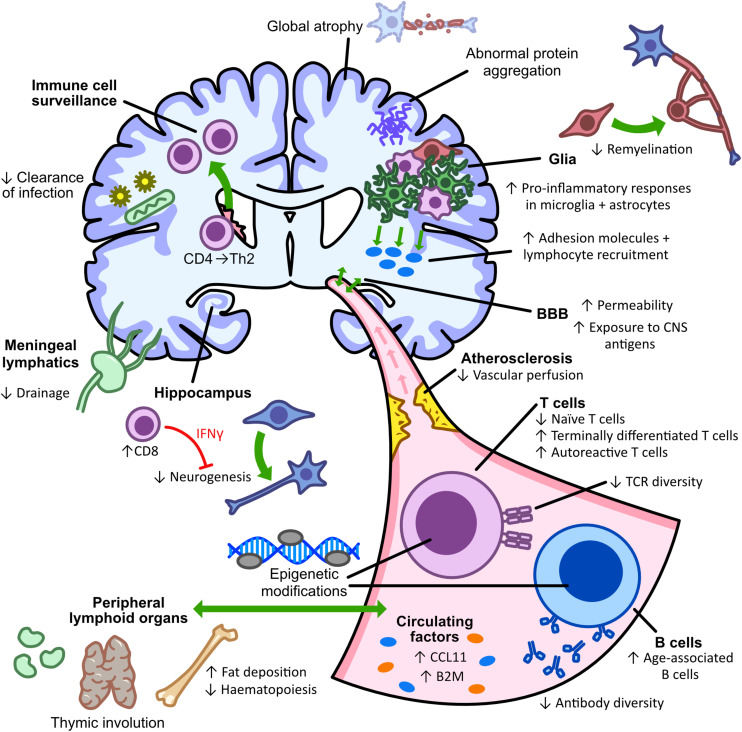
Aging alters CNS-immune interactions in health and disease. Aging is a major risk factor for neurodegeneration that is accompanied by progressive immunosenescence, inflammaging, atrophy, and neuroinflammation. Microglia and astrocytes are thought to respond more to pro-inflammatory cytokines, such as IFNγ and TNFα, and become prone to abnormal inflammatory activation; leading to reduced remyelination and enhanced lymphocytic recruitment. Subsequent aging-related changes in BBB permeability and lymphatic drainage also increase the infiltration of cytotoxic CD8^+^ T cells to the brain, which can inhibit neurogenesis through IFN-γ signaling. In the periphery, processes such as thymic involution and epigenetic modifications similarly alter the number of naïve CD4^+^ T cells and can diminish the antibody repertoire following an increase in age-associated pro-inflammatory B cells. By contributing to poor infection clearance, protein aggregation and altered immune cell surveillance, aging therefore has a detrimental impact on both the immune system and cognitive functioning.

These studies stress the detrimental relationship of the CNS and the adaptive immune system during aging, which modulates the functional connection between the two (summarized in Figure 2). Aging leads to an increase in pro-inflammatory cytokines and a decrease in T cell repertoire and pro-regenerative functions. Therefore, aging tilts the balance in favor of the degenerative role of the adaptive immune system in the CNS at the expense of its regenerative functions.

## Concluding Remarks

This review has detailed the role of the adaptive immune system in both degeneration and regeneration in neurodegenerative diseases. In these diseases, the adaptive immune system is often dysfunctional, with altered levels of immune cells and shifts to a more pro-inflammatory immune profile, both of which are associated with neurodegeneration. Other studies have also shown a neuroprotective and regenerative role for the adaptive immune system in animal models of disease. However, despite research suggesting a greater role for adaptive immunity in progressing neuronal loss, it is debatable whether this is truly due to a greater detrimental role of the adaptive immune system or rather due to the less abundant research in neuroregeneration; a newer and currently growing field. There is also difficulty in establishing a definitive regenerative effect for the adaptive immune system, with many studies failing to distinguish between neuroprotection and neuroregeneration. This may be due to the variety of animal models used to study these diseases, and their limitations in evaluating regeneration.

Interestingly, the normal biological process of aging causes similar dysregulation of the adaptive immune system. As aging is a major risk factor of neurodegenerative diseases, often influencing progression and evolution of disease severity, immunosenescence and the declining function of the CNS may influence neurodegeneration. This is of particular importance when evaluating the dual role of the adaptive immune system in the CNS. The switch of the adaptive immune system between degenerative and regenerative effects may have an environmental or age-associated trigger. This reveals a need to expand research on neurodegenerative diseases into aging animal models, furtherly delving into the opposing sides of adaptive immunity in the CNS. Future research into the mechanisms of the adaptive immune system in neuroregeneration is also required, and whether this regenerative role is lost during disease progression and aging before establishing therapeutics. To date, immunotherapies for neurodegenerative diseases have focused on targeting protein aggregation, however, there may be scope for immunotherapies which regulate the pro-inflammatory profiles seen in disease and aging, or immunotherapies focused on boosting the neuroprotective and neuroregenerative function of the adaptive immune system.

## Author Contributions

KM wrote the manuscript. CM designed and created the figures. KM, JAW, CM, FJR, and AdlF contributed to literature research and edited of the manuscript. All authors contributed to the article and approved the submitted version.

## Conflict of Interest

The authors declare that the research was conducted in the absence of any commercial or financial relationships that could be construed as a potential conflict of interest.
